# Landscape of biomolecular condensates in heat stress responses

**DOI:** 10.3389/fpls.2022.1032045

**Published:** 2022-10-06

**Authors:** Violeta Londoño Vélez, Fatema Alquraish, Ibrahim Tarbiyyah, Fareena Rafique, Duruo Mao, Monika Chodasiewicz

**Affiliations:** Center for Desert Agriculture, Biological and Environmental Science and Engineering Division, King Abdullah University of Science and Technology (KAUST), Thuwal, Saudi Arabia

**Keywords:** biomolecular condensates, LLPS, heat stress response, stress granules, signaling

## Abstract

High temperature is one of the abiotic stresses that plants face and acts as a major constraint on crop production and food security. Plants have evolved several mechanisms to overcome challenging environments and respond to internal and external stimuli. One significant mechanism is the formation of biomolecular condensates driven by liquid–liquid phase separation. Biomolecular condensates have received much attention in the past decade, especially with regard to how plants perceive temperature fluctuations and their involvement in stress response and tolerance. In this review, we compile and discuss examples of plant biomolecular condensates regarding their composition, localization, and functions triggered by exposure to heat. Bioinformatic tools can be exploited to predict heat-induced biomolecular condensates. As the field of biomolecular condensates has emerged in the study of plants, many intriguing questions have arisen that have yet to be solved. Increased knowledge of biomolecular condensates will help in securing crop production and overcoming limitations caused by heat stress.

## 1 Introduction

Due to anthropogenic activities, such as transportation, electricity production, and other industries, the world is facing an observable change in climate (IPCC, 2007). The average world temperature has significantly increased since the 1980s, according to data from NASA’s Goddard Institute for Space Studies (GISS; Janni et al., 2020). The global air temperature has been forecast to increase by 1.8−4 C by the end of the 21st century compared to current levels, increasing by approximately 0.2°C per decade (Bita and Gerats, 2013; Hasanuzzaman et al., 2013). This increase in global temperature poses an unfavorable environment for plants and may considerably affect their growth and yield (Raza et al., 2019). High temperatures can cause significant damage at the molecular level, leading to alterations in the development and growth of plants (Chaudhry and Sidhu, 2022). Rising temperatures have been suggested as a major limiting factor for crop yield and production worldwide. Africa, Asia, and the Middle East may encounter a drastic decrease in crop yield and production, reaching up to 35% if the temperature increases by 3–4°C in these regions (Ortiz et al., 2008). By 2050, the world population is predicted to rise to over nine billion, and the demand for food will increase accordingly. It has been suggested that world food production must increase by 70% to fulfill the needs of the population by 2050 (Bita and Gerats, 2013).

Plants face many biotic and abiotic stresses, and heat stress is a major plant stress. Heat stress is defined as exposure to elevated temperatures above a certain threshold for a sufficient amount of time to cause irreversible harmful effects in plants at the morphological, biochemical, and physiological levels (Wahid et al., 2007). Heat stress impairs several growth stages and metabolic processes at the cellular level, ultimately leading to a loss of yield quantity/quality or death (Bita and Gerats, 2013; Ahmad et al., 2022).

At the morphological and physiological levels, heat stress causes several types of damage, such as inhibition of shoot and root growth, leaf senescence, leaf scorching and sunburn, fruit damage (discoloration), changes in osmotic pressure, decreased photosynthesis, hormonal changes, and other injuries (Hu et al., 2020; Ahmad et al., 2022). Researchers have always been interested in how plants perceive heat at the molecular level and translate this stress into functional cellular signals. Numerous macromolecules have been found to be involved in sensing heat stress *via* a process called thermosensing (Jung et al., 2020; Lin et al., 2020). Thermosensors are considered the first vital players in plant thermotolerance, but its work is not only limited to harsh conditions. Once thermosensors are activated by an external stimulus, they induce biochemical, biophysical, physiological, and morphological changes within the plant to maintain maximal productivity.

Upon the exposure of the plant to heat stress, the plant activates heat-shock response. This response is associated with an adjustment in the fluidity of the plasma membrane of plants when the embedded thermosensors is activated by heat (Guihur et al., 2022b). Biochemically, the concentration of imminent signaling secondary messengers is considerably increased including calcium ions (Ca^2+^) and reactive oxygen species (ROS). Ca^2+^ and ROS collaborate together with other proteins including, respiratory burst oxidase homolog (RBOH), nicotinamide adenine dinucleotide phosphate (NADPH) oxidase, and calcium-dependent protein kinase (CDPK) and many others for achieving adaptation to heat-shock (Qari and Tarbiyyah, 2021). This collaboration between secondary messengers aid in initiation genetic regulation and accumulation of crucially identified thermotolerance molecules such as, heat shock proteins (HSP) as molecular chaperons, heat shock factors (HSF) as signaling molecules and other heat-induced metabolites. How plants sense and respond to heat stress has been extensively reviewed (Guihur et al., 2022a; Guihur et al., 2022b). Research on plants demonstrated that many thermosensors are essential to trigger adaptive responses that balance cellular homoeostasis and limit the detrimental effects of heat stress. Intensively researched examples of thermosensors in plants include phytochrome B (PhyB), phototropins, cytochromes, and UVR8 (Legris et al., 2016; Fujii et al., 2017). Moreover, the basic helix–loop–helix transcription factor PIF4, a well-known thermosensor in *Arabidopsis*, plays a pivotal role in sensing heat stress signals and orchestrating transcriptional regulation that consequently leads to elongation responses (Han et al., 2019). Also, some thermosensors, such as ELF3, is shown to be sequestered and localized in transient aggregated bodies that will be discussed later in this review.

The thermosensing mechanism in plants translates environmental cues into morphological changes that ultimately reduce the potentially harmful impact of heat stress (Quint et al., 2016; Lin et al., 2020; Ludwig et al., 2021). Depending on the strength of heat stress, plants initiate several morphological changes that facilitate adaptation and acclimation to this stress in a process known as thermomorphogenesis (Quint et al., 2016; Ludwig et al., 2021). At the earliest stages of thermomorphogenic activity, petiole and hypocotyl elongation have been observed in *Arabidopsis* seedlings exposed to heat stress. Petiole and hypocotyl elongation coupled with upward elongation of the rosette leaves and cotyledons, a process known as hyponasty, allows plants to reduce direct heat flux received from the sun and allows cool breezes to reach the stressed leaves; this contributes to the plants’ acclimation to elevated temperatures (Quint et al., 2016; Lin et al., 2020). The outcome of thermomorphogenic activity is dependent on plants’ thermosensing mechanisms.

Intensive research on stress response and adaptation in plants has revealed the formation of spatiotemporal enigmatic structures as another response mechanism at the cellular level (Chodasiewicz et al., 2020; Emenecker et al., 2021; Tong et al., 2022). In the literature, these structures have been referred to as biomolecular condensates, aggregates, assemblies, and membraneless organelles (MLOs), among many other terms. Condensate formation is an evolutionarily conserved phenomenon observed across kingdoms. These condensates were also found in both yeast and mammalian cells (Protter and Parker, 2016). Also, animals cells share many biomolecular condensates with plant cell (Emenecker et al., 2020). Aside from membrane-bound organelles, cells have the ability to form membraneless microcompartments that spatially and temporally concentrate proteins, nucleic acids, and metabolites. Biomolecular condensates have been observed in the nucleus, cytoplasm, and chloroplast (Ouyang et al., 2020; Zavaliev et al., 2020). Some of these condensates are constitutive, whereas others are more specific to certain cell types, environmental cues, and stimuli (Emenecker et al., 2020; Kim et al., 2021). The condensates also vary in terms of composition, architecture, dynamics, and localization. The condensates’ components have been studied using different experimental approaches such as mass spectrometry and RNA sequencing of isolated condensates (Youn et al., 2019). These differences may serve as indicators of certain condensates’ biological relevance and functions.

Biomolecular condensates are attracting considerable interest, as they play significant roles in various biological processes and pathways and could also work as a form of triage for molecules under stress conditions or as a means of molecule sorting and transportation (Hayes et al., 2020; Ouyang et al., 2020; Peran and Mittag, 2020; Emenecker et al., 2021). Interestingly, it has been proven that these aggregates are involved in several crucial biological functions of plants, including hormone signaling, polyadenylation, immune responses, processing noncoding RNAs, and temperature sensing (Fang et al., 2019; Powers et al., 2019; Chodasiewicz et al., 2020). Moreover, an increasing amount of evidence supports the notion that the formation of MLOs is facilitated by liquid–liquid phase separation (LLPS) or a similar mechanism (Emenecker et al., 2021). In this review, we will examine how LLPS contributes to biomolecular condensate formation. Then, we will explore some of the plant-specific condensates and illustrate their involvement in the heat stress response. Finally, we will introduce bioinformatics tools that can predict which proteins are recruited to biomolecular condensates *via* LLPS.

## 2 LLPS driving condensate formation

The process of LLPS is a reversible cellular mechanism that occurs in a very dynamic manner and promotes the formation of two distinct phases: a dense phase and a dilute phase (Emenecker et al., 2020). Due to its efficient dynamics, LLPS is assumed to enable biomolecular condensates in plants to assemble and disassemble in a very intricate manner as required by the cell, including heat stress (Posey et al., 2018; Peran and Mittag, 2020; Emenecker et al., 2021). The mechanism of LLPS critically depends on i) the concentrations of macromolecules, such as proteins, DNA, and RNA; ii) cellular conditions, including pH, temperature, and salt concentration; and iii) post-translational modifications (PTMs), such as phosphorylation, glycosylation, methylation, and acetylation (Brangwynne et al., 2015; Bah and Forman-Kay, 2016; Banani et al., 2017; Emenecker et al., 2021; Sanchez-Burgos et al., 2022).

A critical characteristic of proteins involved in the LLPS mechanism is the presence of intrinsically disordered regions (IDRs), and proteins containing IDRs are called intrinsically disordered proteins (IDPs). Nearly one-third of the proteins in the eukaryotic proteome contain IDRs (Xu et al., 2021). The amino acid composition of IDRs prevents the proper folding of IDPs due to their unstructured propensity (Babu et al., 2011). The IDRs of IDPs typically have signature amino acid sequences that allow them to function in cellular signal transduction, intracellular transport, protein degradation, post-transcriptional modification, transcriptional and translational regulation, protein phosphorylation, and chaperoning other proteins and RNA molecules (Wright and Dyson, 2015). Furthermore, IDRs induce the assembly of biomolecular condensates through weak intermolecular and/or intramolecular interactions that promote LLPS, including hydrophobic contacts, electrostatic bonds, π–π stacking, and cation–π bonds. These weak, transient interactions are among the major features that allow biomolecular condensates to form in a unique, dynamic manner (Banani et al., 2017; Peran and Mittag, 2020; Kim et al., 2021; Xu et al., 2021).

Studies have confirmed that RNA-binding proteins (RBPs) and RNAs are essential components for the stability and architecture of many biomolecular condensates. More specifically, RBPs are multidomain proteins that, like other proteins involved in LLPS, are enriched with IDRs that facilitate the multivalency needed for dynamic, reversible LLPS (Järvelin et al., 2016; Van Treeck and Parker, 2018; Corley et al., 2020). By behaving and functioning as linkers between protiens’ binding domains, IDRs often arrange the RNA-binding domains (RBDs) found in RBPs (Ottoz and Berchowitz, 2020). These linkers are not typically able to create single, well-folded structures and play a significant role in RNA–protein interaction by regulating the spatiotemporal availability and concentration of RBDs, such as the RNA recognition motif (RRM), hnRNP K homology (KH), and zinc finger (ZF) domains (Lunde et al., 2007). Furthermore, RBPs are enriched with low-complexity domains (LCDs), which are considered a type of IDRs. The LCDs found in RBPs are known to be engaged in the dynamic formation of biomolecular condensates (Xu et al., 2021). Studies have shown that the LCDs in RBPs contain an amino acid sequence conserved among eukaryotes that includes tandem repeats, such as polyasparagine (polyN) and polyglutamine (polyQ; Altmeyer et al., 2015; Jung et al., 2020). Additionally, biomolecular condensates are enriched with LCDs known as prion-like domains (PrLDs; Gomes and Shorter, 2019). For example, Jung et al. (2020) identified the presence of a PrLD with a continuous stretch of polyQ repeats in an RBP associated with biomolecular condensates.

Plants exhibit many physiological changes at the cellular level in response to stresses, such as changes in pH, temperature, or salt concentration (Kader et al., 2007). As mentioned previously, LLPS formation is critically dependent on cellular conditions, such as pH, temperature, salt concentration, and PTMs. Many biomolecular condensates are assumed to form through LLPS in plant cells, including the nucleolus, nuclear speckles, Cajal bodies, photobodies, dicing bodies, processing bodies (P-bodies), DNA damage foci, and stress granules (SGs). In terms of heat stress and LLPS in mammalian cells, SGs—a type of biomolecular condensate also found in plants and yeast—are assembled after exposure to endogenous and exogenous stresses, such as heat shock, oxidative stress, pathogen infection, and DNA damage (Jolly et al., 1999; Molliex et al., 2015; Wheeler et al., 2016; Chen and Liu, 2017; Yang et al., 2020). In other terms, SGs are membraneless bodies that are produced in response to stress conditions and disassemble during the recovery phase (Kedersha and Anderson, 2002). Previous studies have suggested that a potential mechanism by which these assemblies adapt to stress is by creating a suitable atmosphere wherein mRNAs are sequestered and stalled in translation initiation. However, more recent research has shown that not all mRNAs sequestered in SGs are stalled in the preinitiation stage of translation (Mateju et al., 2020). Due to the proposed dynamic propensity of LLPS, these mRNAs resume translation when stress is removed (Ivanov et al., 2019; Matheny et al., 2019). Another type of cytoplasmic biomolecular condensates, known as P-bodies, have also been found to have components that play crucial roles in the plant immune response and temperature sensing (Chantarachot and Bailey-Serres, 2018; Guzikowski et al., 2019; Matheny et al., 2019; Jang et al., 2020).

A change in cellular temperature is one condition associated with the formation of biomolecular condensates, including SGs and P-bodies (Cuevas-Velazquez et al., 2016; Nguyen et al., 2016; Koguchi et al., 2017; Hayes et al., 2020; Ouyang et al., 2020; Tong et al., 2022). For instance, one of the main functions of SGs is to protect RNAs from damage due to environmental stress (Maruri-López et al., 2021). One crucial component of SG-associated plant proteins, known as UBP1b, was found to assemble in cytoplasmic entities in response to heat stress exposure in *Arabidopsis.* In particular, UBP1b acts as an mRNA protectant from degradation by interacting with the 3′-UTR of mRNAs during stress (Nguyen et al., 2016; Muleya and Marondedze, 2020). In response to high temperatures, the poly(A)-binding protein Pab1 forms condensates, enabling cells to survive during heat stress (Omkar and Truman, 2022). Additionally, a key regulator of the plant cell cycle during heat stress, known as CDKA1, has been found to remain in a stalled state while sequestered into SGs in *Arabidopsis* (Kosmacz et al., 2019). When temperatures reach normal conditions, the SGs disassemble, allowing for the release of CDKA1 (Kosmacz et al., 2019). These examples support the concept that biomolecular condensate formation is highly associated with cellular and molecular responses to alterations in external temperatures.

## 3 Biomolecular condensates in plants and heat stress response

### Biomolecular condensates in the nucleus

Recently, substantial attention has been paid to nuclear condensates due to their involvement in gene regulation and cellular responses to various stimuli.

#### Nuclear constitutive condensates

Splicing and epigenetic organization are among the mechanisms of gene regulation. Nuclear speckles localize in the interchromatin sites in close proximity to active transcription sites. Prior studies have revealed that nuclear speckles are enriched in splicing factor proteins that facilitate transcriptional regulation (Reddy et al., 2012). Functionally similar bodies called polycomb group (PcG) bodies contain PcG proteins and epigenetic regulators that modify chromatin organization (Pirrotta and Li, 2012).

More recently, studies have indicated that certain biomolecular condensates play pivotal roles in RNA metabolism and biogenesis (Ding and Lozano-Durán, 2020; Reddy et al., 2012; Love et al., 2017; Kalinina et al., 2018; Wu et al., 2019; Ding and Lozano-Durán, 2020). An example of these condensates are Cajal bodies, that consist of proteins, small noncoding RNA, and mRNA. In addition to their role in RNA metabolism and ribonucleoprotein particle (RNP) biogenesis, Cajal bodies may act as modulators of viral infection (James et al., 2010; Shaw et al., 2014; Love et al., 2017; Ding and Lozano-Durán, 2020). However, this involvement is a double-edged sword. Although this involvement can contribute to the host defense mechanism. The nucleolus is a ribonucleoprotein aggregate that functions in ribosomal RNA (rRNA) biosynthesis and ribosome biogenesis, in addition to its emerging role in growth, development, and response to biotic and abiotic stresses (Boisvert et al., 2007; Taliansky et al., 2010; Kalinina et al., 2018; Lafontaine et al., 2020).

A study by Fang et al. (2019) revealed that FLOWERING LOCUS CA (FCA)—a nuclear RBP that participates in RNA processing—forms nuclear bodies. Along with other proteins, FCA regulates flowering time. Furthermore, FCA condensation is aided by other proteins and acts as an enhancer of the polyadenylation of target genes (Fang et al., 2019).

A variety of plant hormone signaling pathways are involved in the response and acclimation to abiotic stresses (Li et al., 2021). For example, abscisic acid (ABA) regulates the expression of heat shock protein 70 (HSP70) during heat stress (Li et al., 2014; Suzuki et al., 2016). Three transcriptional regulators of ABA—ABA INSENSITIVE5 (ABI5), ABI5-INTERACTING PROTEIN1 (AFP1), and CONSTITUTIVE PHOTOMORPHOGENESIS1 (COP1)—localize to subnuclear condensates in *Arabidopsis* (Lopez-Molina et al., 2003; Lynch et al., 2017). While the functions of these plant-specific condensates in hormone signaling require further investigation, they are highly likely to be key players in the function of ABA in the heat stress response (Emenecker et al., 2020).

#### Nuclear inducible condensates

One example of an inducible condensate is the formation of photobodies in response to the inactivation of CRYPTOCHROME 2 (CRY2) by blue light. Notably, CRY2 forms a complex with RNA methyltransferase, which has the ability to regulate RNA methylation (Wang et al., 2021). In contrast, red light induces the formation of PhyB photobodies through phase separation. PhyB can act as both a light receptor and a temperature sensor. This dual functionality is derived by different mechanisms. Under red light, activated PhyB translocates into the nucleus of plant hypocotyl cells and aggregates to form condensates that aid in signal transduction (Chen et al., 2022). Under high temperatures, PhyB is released from condensates and changes from activated PhyB (Pfr) to its inactive form (Pr). Pr stabilizes a number of transcription factors, such as PIF4, which in turn activate FLOWERING LOCUS T (FT) and accelerate flowering (Kumar et al., 2012; Hahm et al., 2020; Lamers et al., 2020).

**Figure 1:**
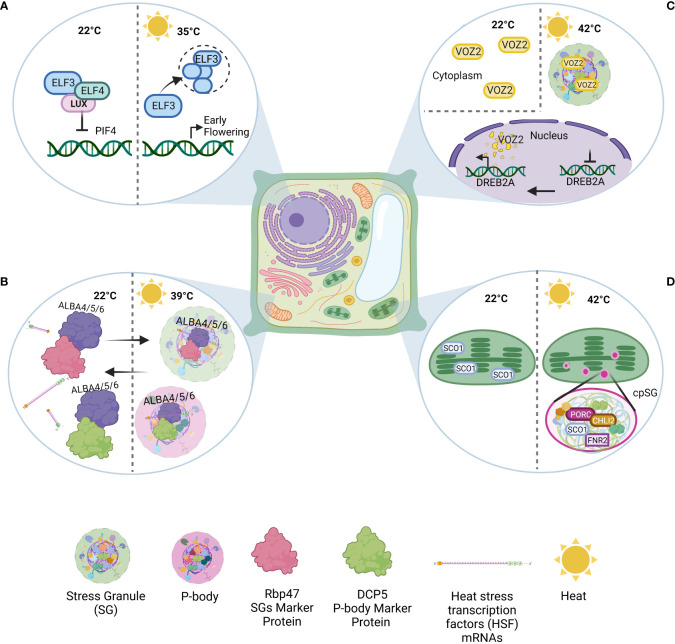
Examples of condensates formed in *Arabidopsis thaliana* under heat. **(A)** ELF3 nuclear condensate: Under normal conditions, ELF3, ELF4, and LUX ARRHYTHMO (LUX) form the EVENING COMPLEX (EC), a repressor of various genes. In response to high temperatures (warming, 35°C), ELF3 condensation causes EC disassociation, target gene activation, and subsequent early flowering. **(B)** ALBA-SGs: Under normal conditions, ALBA4, ALBA5, and ALBA6 can interact with Rbp47 and DCP5. Under heat shock (39°C), ALBA acts as a scaffold to stress granule and P-body assembly. At the same time, ALBA binds and stabilizes HSF mRNAs to protect them from degradation. **(C)** VOZ2-SGs: VOZ2 is dispersed throughout the cytosol under normal conditions. During heat stress (42°C), VOZ2 is transferred to the nucleus, inhibiting certain genes (e.g., DREB2). Meanwhile, in the cytoplasm, VOZ2 colocalizes with SGs and partially with P-bodies. However, nuclear VOZ2 is degraded after two hours. **(D)** cpSGs: Heat stress (42°C) can induce cpSG formation. The proteomics/sequence analysis revealed the presence of 88 proteins in cpSGs (e.g., FNR1, PORC, and CHLI2).

Another example of an inducible condensate is EARLY FLOWERING 3 (ELF3) bodies, which form in response to high temperatures (warming, 35°C) in a PrLD-dependent manner (Hayes et al., 2020). The formation of ELF3 bodies transcriptionally activates a variety of genes that are repressed by the EVENING COMPLEX (EC). The EC consists of ELF3, ELF4, and LUX ARRHYTHMO (LUX). However, ELF3 condensates in response to high temperatures, resulting in the disassociation of the EC and thus activating target genes (**Figure 1A**). The thermosensitivity of ELF3 governs this regulation (Jung et al., 2020). This temperature response positively correlates with the polyQ length in the PrLD. This was further proven by investigating the PrLDs of other plants that are evolutionarily adapted to different climate conditions. For example, *Solanum tuberosumas* inhabits temperate climates and has a shorter PrLD, whereas *Brachypodium distachyon* lacks a PrLD in EFL3 (Jung et al., 2020).

Inducible biomolecular condensates can form not only in response to external stimuli but also in response to internal stimuli. For example, DNA damage triggers the assembly of DNA repair foci. Several proteins involved in DNA repair machinery are recruited and concentrated at the site of the damage. While some proteins can act directly in the repair mechanism, others may serve as recruiters to repair machinery (Rothkamm et al., 2015; Hirakawa and Matsunaga, 2019).

The precise mechanism by which LLPS participates in stress signal transduction and gene regulation remains elusive.

### Biomolecular condensates in the cytoplasm

Several plant processes that occur in the cytoplasm—such as hormone signaling, polyadenylation, and immune responses—may partially or fully occur in biomolecular condensates (Fang et al., 2019; Powers et al., 2019).

#### Cytoplasmic constitutive condensates

To achieve optimized growth and development, it is crucial that plants maintain a balance between mRNA decay, translation, and sequestration (Jang et al., 2020). Being unstable and mobile in nature, RNA allows for both the sequence-dependent and independent regulation of gene expression. The diversity of eukaryotic mRNA decay pathways demonstrates the importance of decreasing transcript levels to regulate gene expression (Maldonado-Bonilla, 2014). As mentioned previously, the cytoplasm of eukaryotic cells contains RNA–protein assemblies, including P-bodies and SGs. The mRNA turnover pathway begins with deadenylation, followed by decapping and decay in the 5`–3` direction, and this exonucleolytic activity occurs in cytoplasmic foci called P-bodies (Sheth and Parker, 2003). However, it has been discovered that the presence of P-bodies is not required for mRNA degradation (Eulalio et al., 2007). Furthermore, the origination of P-bodies does not appear to require specific conditions, as they can be detected in most somatic cells under normal cellular conditions (Bashkirov et al., 1997).

The core components and prominent markers of P-bodies include 5`-exoribonuclease, deadenylases, and the subunits of decapping enzymes (DCPs), such as DCP1, DCP2, and Hed1S (van Dijk et al., 2002). The mRNAs in P-bodies lack poly-A tails, as the removal of poly-A tails appears to be an early step in the formation of P-bodies or mRNAs recruited to preexisting P-bodies, confirming that these mRNAs are specifically primed for decay (Zheng et al., 2008). However, one study reported that mRNAs entrapped in P-bodies can be recycled and translocated to polysomes (Brengues et al., 2005). Despite the association of most proteins present in P-bodies with mRNA degradation, live imaging has shown no mRNA decay events in P-bodies. Furthermore, no truncated mRNAs were found in P-bodies, and the inhibition of DCP2 and RNAase which are the main components of P-bodies was observed in an *in vitro* reconstituted LLP system (Horvathova et al., 2017; Schütz et al., 2017).

Post-embryonic lethality is observed in *Arabidopsis* after mutation in most of the components of P-bodies, suggesting that the core components of P-bodies are required for developmental transition during an early developmental program. Transcriptomic and translatomic analysis revealed that P-bodies sequester and defer the translation of thousands of mRNA when the seedlings of wild type and *Arabidopsis* mutant defective in DECAPPING 5 (DCP5), dcp5-1 were compared (Jang et al., 2019; Jang et al., 2020). Additionally, shifts in the sizes, concentrations, and properties of P-bodies were reported to occur during stress in a recent study. In the absence of stress, P-bodies mostly consisted of poorly translated mRNAs, whereas longer RNAs were entrapped in the P-bodies of stressed cells (Matheny et al., 2019). Finally, the knockout or overexpression of certain core proteins in P-bodies alters plant responses to various forms of environmental stress (Jang et al., 2020).

Along with DCP proteins, tandem-CCH-type Zinc Finger (TZF) proteins have been found to be present in P-bodies (Pomeranz et al., 2010). In plants, TZF proteins play important roles in several developmental and environmental responses by controlling mRNA turnover (Li and Thomas, 1998). In *Arabidopsis thaliana*, AtTZF1 has been shown to colocalize with markers of P-bodies (AGO1, DCP2, and XRN4) under non-stress conditions. Additionally, AtTZF1 colocalized with the SG marker PABP8 when transformed maize protoplasts were subjected to heat shock at 42°C for 30 mins., proving that dynamic subcellular localization of AtTZF1 occurs in P-bodies and SGs. This enhances plant stress tolerance in a gibberellin (GA)-dependent and ABA-dependent manner by differential regulation of the genes involved in the metabolism of these hormones. For example, AtTZF4/5 acts as a positive regulator of ABA biosynthesis while upregulating GA-catabolic genes, thus enhancing the plant’s response to abiotic stresses (Pomeranz et al., 2010). Similarly, OsTZF7 is upregulated and sequestered in P-bodies/SGs during drought conditions. In the nucleus, chromosome region maintenance (CRM1) binds to nuclear export signals (NES) within the C-terminus of OsTZF7 and mediates its nuclear export. Once out of the nucleus, OsTZF7 binds to the AU-rich motifs (AREs) within the 3` untranslated regions of target mRNAs to regulate their degradation (Guo et al., 2022). Despite our increasing knowledge of P-bodies, we still do not fully understand the processes of mRNA decay and sequestration within P-bodies.

#### Cytoplasmic inducible condensates

The metabolism of mRNAs is important for growth, development, and regulation of the stress response. These actions require the assembly of mRNA–ribonucleoprotein (mRNP) complexes (Chantarachot and Bailey-Serres, 2018). In the 1960s, granular structures comprised mostly of HSPs were observed in chicken embryo fibroblasts and HeLa cells exposed to heat stress (Collier and Schlesinger, 1986). Then, cytoplasmic foci (called SGs) containing untranslated mRNAs were reported to be produced by mammals in response to stress. These SGs formed due to the blocking of translation initiation by the phosphorylation of eukaryotic initiation factor 2α (elf2α). The primary components of these mammalian SGs were found to be the poly-A protein and mRNA. (Kedersha et al., 1999). Analogous structures were found in yeast upon the application of mild heat stress in combination with other stresses, which caused bulk translation repression (Yamamoto and Izawa, 2013). Plant SGs share the same structure (i.e., stable RNP cores with diffusible shells) as mammalian and yeast SGs. Yeast SGs contain orthologues of mammalian SG components (TIA-R, TIA-1, and Ataxin-2), such as PUB1, NGR-1, and PAB1. As in mammals, yeast SG assembly is blocked by cycloheximide and promoted by the phosphorylation of elF2α (Buchan et al., 2008). Much research on the assembly and disassembly of SGs has been carried out in mammals and yeast, and alterations in these processes have been linked to degenerative diseases (Ramaswami et al., 2013).

In plant cells, three cytoplasmic stress-induced granules were detected using immunofluorescence labelling and characterized into different entities according to their composition (heat SGs, SGs, and P-bodies; Weber et al., 2008). The structure of an SG consists of a stable core and a more dynamic shell, each characterized by different protein/metabolite/RNA compositions. The heterogeneous composition of SGs varies according to the type of stress faced by the plant (Kosmacz et al., 2018). These foci function by helping plants survive stressful conditions through different means, including i) protecting mRNAs from degradation, ii) exporting specific mRNAs for decay, and iii) protecting proteins from unfolding and degradation (Anderson and Kedersha, 2006).

The core proteins of SGs that drive LLPS contain oligomerization domains, including IDRs, PrLDs, and RBDs (Sanders et al., 2020). These domains are majorly responsible for the assembly of stress-induced granules. A time-course analysis of a single molecule revealed that the first step in the formation of SGs is nucleation (Protter and Parker, 2016). This step is dependent on the interaction domains of core proteins and mRNAs present in the stable cores of SGs (Ditlev et al., 2018). The most exceptional role in this step is played by mRNAs, as they are also involved in controlling the transcriptomes and proteomes of SGs. *In vitro* studies have shown that SGs assemble when mRNAs interact within themselves through Watson–Crick base pairing, helical stacking, and non-canonical base pairing, aiding the stability and morphology of SGs (Van Treeck et al., 2018). The core proteins of SGs (Rbp45 and Rbp47) have been found to drive LLPS and ultimately the assembly of SGs. Recently, Rbp47b interactome data from *A. thaliana* SGs revealed the presence of proteins in the core encoding PrLDs, ATPase and RNA binding domains. In addition, the homo-oligomerization and hetero-oligomerization of proteins is a crucial factor in the nucleation step of SG assembly. Kosmacz et al. (2018) also stipulated the role of a significant small molecule 2’,3’-cAMP in the *in vitro* self-oligomerization of Rbp47b in SGs. This molecule was further proven to play an important role in the stress response (Chodasiewicz et al., 2022). Additionally, RNAs play a significant role in the next phase (i.e., the core growth process). The specific configuration of mRNA can expose certain sequences, allowing for their interaction with other RNAs and RBPs within SGs (Langdon et al., 2018). Once the core is established, RBPs recruit other proteins containing IDRs to ease shell growth. These proteins are stress-dependent, cell-dependent, and organism-dependent and lack RNA-binding capabilities (Markmiller et al., 2018).

One study disclosed that the threshold temperature that is needed for the initiation of SG formation is 34°C. The authors also confirmed the role of actin filaments in the long-distance movement of SGs in *Arabidopsis* cells (Hamada et al., 2018). In *Arabidopsis*, using ANGUSTIFOLIA (AN) as bait, RBP Rbp47b and OLIGOURIDYLATE BINDING PROTEIN (UBP1b) were determined to be key players in the assembly of SGs (Bhasin and Hülskamp, 2017). In UBP1b-overexpressed lines, heat stress-induced SGs enhanced tolerance to heat stress. Furthermore, DnaJ heat shock protein, a stress-associated protein (AtSAP3), two candidate UBP1b mRNA targets, and 115 other heat stress-inducible genes were identified to have increased expression and stability under heat stress. This was further confirmed by *ubp1b* mutant studies, in which these plants were found to be heat sensitive (Nguyen et al., 2016).

It is commonly accepted that SGs contain stalled mRNA complexed with translation initiation factors (eIF4E, eIF4G, eIF4A, eIF3, and eIF2), proteins with ATPase activity, and chaperones with 40S ribosomal subunits. A recent study identified the presence of 118 proteins in heat induced SGs of *Arabidopsis* by using Rbp47b as bait where 25% exhibited high homology to SG-associated proteins across all kingdoms (Kosmacz et al., 2019).

Furthermore, over the years, scientists have identified many RNA-binding proteins [e.g., Rbp47, poly(A)-binding proteins] as SG markers in plants, suggesting that they play essential roles in SG formation and plant stress resistance. Another two RBPs in *Arabidopsis*, named RBGD2/4 (RNA-binding glycine-rich D2/4), are recruited into SGs under heat stress (Zhu et al., 2022). In the LCD of RBGD2/4, the tyrosine residue array (TRA) is necessary and responsible for LLPS and the formation of SG in heat stress. It has been found that mutations in the TRA can disrupt the protein ability to undergo LLPS and can directly impair their strength in heat stress defense. This provides robust evidence that protein LLPS can be a heat stress-resistant strategy in plants. Furthermore, RBGD2/4 can undergo phase separation both *in vivo* and *in vitro* under heat, whereas this only occurs *in vitro* under cold stress (Zhu et al., 2022). This suggests a relationship between stress-induced condensate behavior and the corresponding stress, and it may be due to the fact that different stresses can induce different cellular environments.

Other DNA/RNA-binding proteins include ACETYLATION LOWERS BINDING AFFINITY (ALBA) proteins, which are well studied in archaea and protozoan parasites. Scientists have also identified six types of ALBA proteins in *Arabidopsis*, of which ALBA4, ALBA5, and ALBA6 especially help plants fight heat stress (Tong et al., 2022). Under normal conditions, ALBA proteins can interact with many SG and P-body components, including DCP5 and Rbp47. This suggests the presence of preexisting interactions, which may help in the rapid assembly of SGs and P-bodies under stress. However, under heat shock (39°C) conditions specifically, the three before mentioned ALBA proteins can localize into SGs and P-bodies by interacting with both SG and P-body marker proteins, playing a scaffolding role in SG and P-body assembly (**Figure 1B**). These findings indicate a high potential for the *stress-specific* composition of stress-induced condensates. Furthermore, scientists have found that under heat, these three ALBA proteins can bind and stabilize heat stress transcription factor (HSF) mRNA. In *alba4/5/6* mutants, HSF mRNA stability and abundances are decreased, while total poly(A) mRNAs remain unchanged. These insights suggest that specific RNA-binding proteins may only be responsible for corresponding poly(A) mRNAs under heat stress (Tong et al., 2022).

The disassembly of SGs is a reversible process compared to the assembly of SGs, with shell diffusing occurring first, followed by core dissipation (Maruri-López et al., 2021). It has been shown that conditions that interfere with or perturb the interactions between IDRs are sufficient to dissipate the shell. A 30% reduction in the sizes of SCO1-GFP foci in cpSGs was observed after treatment with 1,6-hexanediol, confirming the statement above that shell diffuses first in the disassembly process (Chodasiewicz et al., 2020). During recovery from heat shock in *A. thaliana*, HSP70 and HSP101 proteins are recruited for dissolution of the SG core, as shown by the hsp101 knockout mutant still harboring SGs even after relieving the stress condition. (Cherkasov et al., 2013; Merret et al., 2017; McLoughlin et al., 2019). These proteins are later redistributed in the cytoplasm as recovery from heat stress continues (Cherkasov et al., 2013; Merret et al., 2017; McLoughlin et al., 2019). Heat shock proteins play an important role in the dispersal of condensates in other organisms as well. Heat shock at 42°C leads to the formation of Pab1 condensates in budding yeast (Wallace et al., 2015). These condensates disperse as soon as the cells recover from the stressed environment. The protein content of these condensates return to their pre-stress soluble form without degradation with the help of HSP104, HSP70, and type II Hsp40 Sis1. These chaperones are responsible for the complete and quick dispersal of Pab1 condensates *in vitro* (Yoo et al., 2022). These results are in accordance with the previous studies where the deletion of Hsp104, Hsp70, or Hsp40 delayed the dispersal of SGs marked by Pab1 *in vivo* (Cherkasov et al., 2013). HSP chaperones have a prominent role in producing heat-resistant crops by sensing high temperature leading to the onset of plant thermotolerance, their types and roles are extensively reviewed in (Guihur et al., 2022b).

Stress can trigger many changes in the cell. Under heat stress, transcriptional regulation cascades cause global translation inhibition. This inhibition triggers the formation of SGs, which are involved in post-transcriptional regulation under various stress conditions. The VASCULAR PLANT ONE ZINC FINGER 2 (VOZ2) is another protein that takes part in heat tolerance *via* biomolecular condensates (Koguchi et al., 2017). This protein acts as a transcriptional repressor of the DEHYDRATION-RESPONSIVE ELEMENT-BINDING PROTEIN 2A (DREB2A), an important transcription factor that activates drought-responsive and heat stress-responsive genes. Under normal conditions, VOZ2 is localized in the cytoplasm. Under heat stress (42°C), VOZ2 is transferred to the nucleus and colocalizes with SGs and partially with P-bodies in the cytoplasm (**Figure 1C**). The nuclear VOZ2 is degraded after two hours of high-temperature incubation, and cytoplasmic VOZ2 sequestration by SGs under high-temperature conditions prevents VOZ2 transfer to the nucleus. Therefore, VOZ2 sequestration and consequent DREB2A upregulation increase heat resistance (Koguchi et al., 2017).

There are other examples of cytoplasmic condensates that form in response to other stimuli, such as LATE EMBRYOGENESIS ABUNDANT (LEA) and AUXIN RESPONSE FACTOR (ARF) condensates (Powers et al., 2019; Dirk et al., 2020). In particular, NONEXPRESSOR OF PATHOGENESIS-RELATED GENES 1 (NPR1) condensates referred to as salicylic acid (SA)-induced NPR1 condensates (SINCs; Zavaliev et al., 2020) appear due to the cellular redox change triggered by SA, causing conformational changes in the NPR1 (Tada et al., 2008). During effector-triggered immunity (ETI), pathogen effectors activate programmed cell death concurrently, and an increase in SA causes the NPR1 homo-oligomers in the cytoplasm to dissociate into monomers, which are released into the nucleus (Cui et al., 2015). In the nucleus, NRP1 is SUMOlyated for its transcription cofactor activity and promotes its degradation by the NPR3/4-CRL3 complex to remove its inhibitory effect on ETI (Mittag and Strader, 2020). Notably, NPR1 promotes cell survival by sequestering and degrading the proteins involved in cell death in the cytoplasm. A study by Zavaliev et al. (2020) confirmed that cell survival by NPR1 occurs not only in pathogen-triggered cell death but also in heat shock, oxidative, and DNA damage responses. This study revealed the role of SA in suppressing cell death triggered by heat shock in WT plants but not in *npr1-2* mutant plants, proving the hypothesis that the NPR1 protein is responsible for regulating the stress response by sequestering stress-related proteins in SINCS.

### Biomolecular condensates in the chloroplast

The presence of biomolecular condensates is not limited to the nucleus or cytoplasm. The chloroplast, which serves as a photosynthetic hub, also hosts several biomolecular condensates. It would be reasonable to expect that chloroplast condensates may assist in photosynthesis and enhance its efficiency. For example, this may occur through the condensation of RIBULOSE-1,5-BISPHOSPHATE CARBOXYLASE/OXYGENASE (RubisCO) within the pyrenoid matrix in green algae and some lower plant species. RubisCO is one of the key enzymes in the carbon cycle that concentrates CO_2_ fixation, leading to increased photosynthesis and biomass (Mackinder et al., 2017; Li et al., 2020; Barrett et al., 2021). Moreover, Atkinson et al. (2020) proved that RubisCo can condensate in *Arabidopsis* chloroplasts when RubisCo and the linker protein ESSENTIAL PYRENOID COMPONENT 1 (EPYC1) are expressed.

Furthermore, Ouyang et al. (2020) were among the first to reveal the transportation and sorting capabilities of one type of chloroplast condensates, a pair of ankyrin repeat proteins called STAUROSPORINE AND TEMPERATURE SENSITIVITIES 1 AND 2 (STT1 and STT2) bodies. These STT bodies recognize and act as cargo transporting proteins from the chloroplast envelope to the thylakoid membrane (Ouyang et al., 2020; Zheng et al., 2021).

Contrary to previous belief, SGs are not exclusive to the cytoplasm. In 2008, Uniacke and Zerges (2008) revealed that oxidative stress can induce the formation of chloroplastic SGs (cpSGs) in a single-cell green alga. More than a decade later, another group discovered the formation of heat-induced (42°C) cpSGs in *Arabidopsis* chloroplasts (**Figure 1D**; Chodasiewicz et al., 2020). These results suggest that cpSGs may play a role in the stress-induced regulation of plastidial machinery and the stress response (Chodasiewicz et al., 2020). However, further experiments are needed to thoroughly elucidate cpSG functions. A recent report focused on the combined use of *A. thaliana* and *C. reinhardtii* as model organisms to uncover the role of cpSGs (Rafique et al., 2022).

## 4 Bioinformatic tools for the analysis of heat stress-induced condensates

Over the decades, scientists have identified many condensates formed *via* LLPS, most of which contain LCDs. Among the various types of LCDs, PrLDs play an outstanding role in supporting proteins undergoing LLPS. There are other LCDs, such as polyampholytic tracts and elastin-like polypeptides. However, PrLDs are intrinsically disordered and enriched in glycine, glutamine, asparagine, and serine.

A number of bioinformatic tools have emerged to predict the proteins that form LLPS, such as Prion-Like Amino Acid Composition (PLAAC) for predicting PrLDs (Lancaster et al., 2014) or Protein Disorder Prediction (DISOPRED3) for predicting IDRs (Jones and Cozzetto, 2015). Researchers have also put effort into predicting the proteins that form heat stress-induced granules. Based on a previous study on the heat stress-induced recruitment of PrLD proteins into SGs, Iglesias et al. (2021) developed Stress Granules neural network (SGnn), an online web tool designed to predict the proteins recruited to heat stress-induced granules. The predictions made by SGnn are based mainly on aggregation propensity, net charge, and the presence of free cysteines. However, other factors may be responsible for biomolecular condensate formation under heat stress in plants that are unexplored yet. Such factors must be considered in these tools in the future to arrive at more accurate predictions and thus warrant exploration.

## 5 Concluding remarks

Most of our knowledge and protocols are based on mammalian cell work and gene orthologs. Thus, knowledge of biomolecular condensates in plants remains scarce and ambiguous. Nevertheless, in recent years, studies on phase separation and the development of biomolecular condensates in plants have grown and gained popularity. More effort is needed to develop new tools and techniques and to enhance existing methods in the field of plant studies. Further research on the subcellular localization of these biomolecular condensates will provide substantial insight into its biological relevance and function. This knowledge could be exploited to enhance crop performance under challenging environmental conditions.

## Author contributions

VV, FA, These authors contributed equally to this work and share first authorship. IT, FR, DM, and MC contributed to the writing and review of this manuscript. All authors contributed to the article and approved the submitted version.

## Conflict of interest

The authors declare that the research was conducted in the absence of any commercial or financial relationships that could be construed as a potential conflict of interest.

## Publisher’s note

All claims expressed in this article are solely those of the authors and do not necessarily represent those of their affiliated organizations, or those of the publisher, the editors and the reviewers. Any product that may be evaluated in this article, or claim that may be made by its manufacturer, is not guaranteed or endorsed by the publisher.
